# ﻿The larval, pupal and mitogenomic characteristics of *Agrilusadelphinus* Kerremans, 1895 (Coleoptera, Buprestidae) from China

**DOI:** 10.3897/zookeys.1174.105479

**Published:** 2023-08-08

**Authors:** Xuyan Huang, Yujie Gan, Lei Wang, Yanying Xu, Zhonghua Wei, Aimin Shi

**Affiliations:** 1 College of Life Sciences, China West Normal University, Nanchong 637009, China China West Normal University Nanchong China; 2 The Key Laboratory of Southwest China Wildlife Resources Conservation of the Ministry of Education, College of Life Sciences, China West Normal University, Nanchong 637009, China China West Normal University Nanchong China

**Keywords:** Larva, mitogenome, pupa

## Abstract

In this study, the larva and pupa of *Agrilusadelphinus* are described and illustrated. DNA barcoding (*COI* gene) was used to associate the larval and pupal stages with adults based on the maximum-likelihood method. In the resulting phylogenetic tree, species from the same species-group were found to be clustered on a branch with high support value. To better understand *A.adelphinus*, the complete mitochondrial genome of this species was also sequenced and annotated. Comparing this genome to the known mitogenomes of *Agrilus* species, the newly sequenced genome is shorter, with 15,732 bp. However, its whole mitogenome composition and gene orientation were consistent with that of most species of Buprestidae. In the mitogenome of *A.adelphinus*, the ATGATAG sequence was observed between *ATP8* and *ATP6*, which is ATGATAA in other insect mitogenomes. Leu2, Phe, Ile, Gly, and Ser2 were the five most frequently encoded amino acids. The results further prove that DNA barcoding can remove the limitation of traditional taxonomy which cannot identify to species all developmental stages. This study also provides valuable molecular and morphological data for species identification and phylogenetic analyses of the genus *Agrilus*.

## ﻿Introduction

The cosmopolitan genus *Agrilus* Curtis, 1825 (Coleoptera, Buprestidae, Agrilinae) is the largest and most difficult to classify in Buprestidae, with more than 3000 valid species (Jendek and Grebennikov 2011). Among them, some species are invasive exotics (Sydnor et al. 2007; Jendek and Poláková 2014; Bozorov et al. 2019), and few species are pollinators of the plant *Albiziajulibrissin* Durraz., as observed by the authors. In recent years, the taxonomy, distribution, and biology of *Agrilus* species have been updated by Jendek (2016) and Jendek and Nakládal (2019, 2021).

The morphological characteristics of larvae are important information which have received much attention, and some taxonomists have used these data in the study of higher-level phylogenetic relationships (McCabe and Godfrey 1982; Smith 1997; Hart 2000; Michat et al. 2007; Lawrence et al. 2011; Staniec and Pietrykowska-Tudruj 2019; Mahlerová et al. 2021). In the family Buprestidae, Bílý and Volkovitsh (2001, 2003, 2005), Volkovitsh and Bílý (2001, 2015), Volkovitsh et al. (2005), and Bílý et al. (2013) have made significant contributions on the development of larval morphology and its implications for classification. Larvae of *Agrilus* from European part of the former USSR were studied and summarized by Alexeev (1960, 1961, 1981). The life history of some important forest pests in the *Agrilus*, which cause significant economic losses, were studied by various experts (Herms 2002; Haack et al. 2009; Jendek and Grebennikov 2009; Jendek et al. 2015; Hoebeke et al. 2017). In recent years, *Agrilusplanipennis* Fairmaire, 1888 and *A.mali* Matsumura, 1924 became important forest pests in the Palearctic Region, and their life history and other biology were studied. The larvae of *A.planipennis* have been described by Wei et al. (2007), Wang et al. (2010), Chamorro et al. (2012), and Orlova-Bienkowskaja and Bieńkowski (2016), and the life history of *A.mali* was reported by Bozorov et al. (2019). However, the above-mentioned *Agrilus* larvae were identified based on the larvae and adults collected on the same location, which may not be quite accurate, as there could be other undiscovered species of *Agrilus* at those locations.

In recent years, DNA barcoding has been widely used in the identification of species and in phylogenetic analyses (Dezfuli et al. 2002; Hebert et al. 2004; Miller et al. 2005; Evans et al. 2015; Koperski 2019; Pan et al. 2021; Li et al. 2022). Mitogenomic genes have also been used to identify species of *Agrilus*; for example, the problem of the *A.viridis* Kerremans, 1894 complex was partly resolved using the *COI* gene (Bernhard et al. 2005; Pentinsaari et al. 2014; Pellegrino et al. 2017), and the dispersal directions and phylogenetics of *A.mali* were analyzed based on DNA barcoding of 37 species (Bozorov et al. 2019). The larvae and adults of *A.ribesi* Schaefer, 1946 were associated using the *COI* gene (Jendek et al. 2015). The molecular phylogeny of the genus *Agrilus* was first demonstrated by Kelnarova et al. (2019), based on the *COI* and *16S* genes of 100 *Agrilus* species, and an undescribed *Agrilus* species from the Western Palaearctic Region was found in North America based on 759 DNA barcodes (Digirolomo et al. 2019).

In this study, the fragment sequences of the *COI* gene are used to identify the larvae, pupa, and adults of *A.adelphinus*, and the mitogenome of this species is sequenced, annotated, and described.

## ﻿Materials and methods

### ﻿Sampling and specimen examination

**Figure 1.**
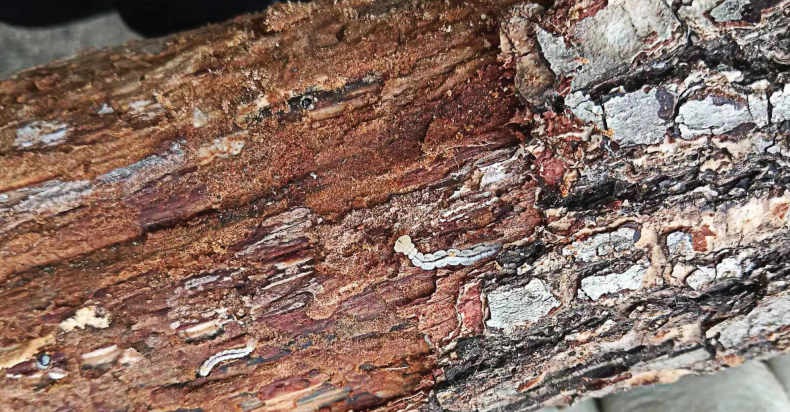
Mature larvae of *Agrilusadelphinus* under the trunk bark of a dead *Quercus* sp.

The specimens were collected in Yanshan Mountains, Hebei Province, China in May 2022. Most adult specimens were collected using insect nets, but a few adults, larvae, and pupae were collected under trunk bark of a dead *Quercus* sp. This tree had bark approximately 10 mm thick (Fig. 1). The specimens were deposited in the College of Life Sciences, China West Normal University (CWNU).

All the specimens were examined using an Olympus SZX10 microscope. Photographs were taken with two different imaging systems: Leica M205FA stereomicroscope equipped with a Leica DFC450 camera and a Canon EOS 9D with a Laowa FF 25 mm F2.8 Ultra Macro 2.5–5× lens. All the figures were edited using Adobe Photoshop CC 2019 to form plates.

The morphological terms used in the descriptions of larva and pupa were introduced by Volkovitsh and Hawkeswood (1990) and Chamorro et al. (2012), which have been widely used in Buprestidae (Bílý and Volkovitsh 2001, 2003, 2005; Volkovitsh and Bílý 2001, 2015; Volkovitsh et al. 2005; Bílý et al. 2013).

### ﻿Molecular analyses

To associate the different stages, the mitogenomic gene *COI* fragment sequences were used for phylogenetic analyses. The genomic DNA of 10 individuals, including five adults, three larvae, and two pupae (Suppl. material 1: table S1), was extracted from head and thorax muscle tissues using the Ezup Column Animal Genomic DNA Purification Kit (Shanghai, China) according to the manufacturer’s instructions. The primers LCO1490 and HCO2198 (Folmer et al. 1994) were used to amplify the fragments of the *COI* gene. The thermal profile was as follows: 94 °C for 2 min; 4 cycles at 94 °C for 30 s, 45 °C for 40 s, and 72 °C for 1 min; followed by the next 34 cycles at 94 °C for 30 s, 51 °C for 40 s, and 72 °C for 1 min; and a final extension at 72 °C for 10 min. The PCR products were sequenced by Sangon Biotech Co. Ltd (Shanghai, China). The sequences were spliced, cut, and proofread using SeqMan v. 7.1.0 under DNASTAR (Burland 2000) and then aligned using PhyloSuite v. 1.2.2 (Zhang et al. 2020).

The mitogenome of *A.adelphinus* was sequenced by Beijing Aoweisen Gene Technology Co. Ltd (Beijing, China). The mitogenomic data were analyzed following the procedures of Huang et al. (2022) and Wei (2022).

## ﻿Results

### ﻿Taxonomy

**Subgenus Quercuagrilus Alexeev, 1998**

***Agrilussulcicollis* species-group**

#### ﻿ Agrilus adelphinus

Taxon classificationAnimaliaColeopteraBuprestidae

Kerremans, 1895

62E8AA01-E7E8-5647-AEF3-340EDD85CBB3

Agrilus adelphinus Kerremans, 1895: 222.
Agrilus egorovi Alexeev, 1989: 480.
Agrilus nigrocoerulans Obenberger, 1924: 39.
Agrilus nonfiedanus Obenberger, 1923: 65.
Agrilus nonfriedi Obenberger, 1914: 49.
Agrilus panhensis Baudon, 1968: 117.

##### Examined specimens.

Adults: 13♂14♀, China: Hebei: Qinhuangdao, 40.3332°N, 119.4090°E, 16-V-2022. Larvae: 13 exs., the same data as adult. Pupae: 5 exs., the same data as adult.

##### Distribution.

China: Hebei, Shanxi, Shandong, Shaanxi, Anhui, Hubei, Guangxi, Sichuan, Yunnan, Xizang; Russia (Far East), Korean Peninsula, Japan.

##### Note.

The adults of *A.adelphinus* appeared in May to August.

##### Description of larva

**Figure 2.**
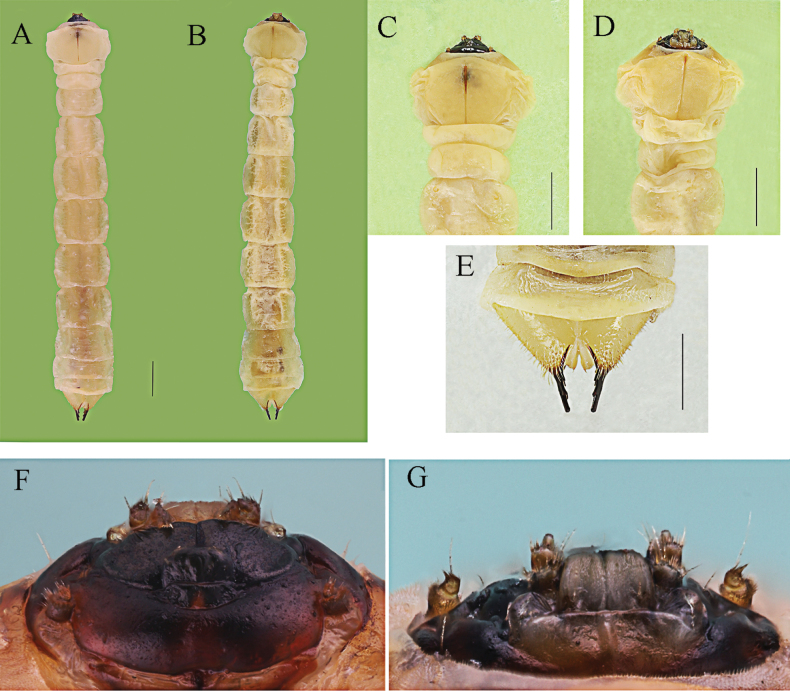
Larva *of Agrilusadelphinus***A, C** dorsal view **B, D** ventral view **E** terminal processes in dorsal view **F, G** mouthparts in dorsal view and ventral view, respectively. Scale bars: 1 mm (**A–E**).

**(Fig. 2).** Body length 9–17 mm; widest in prothorax, 1–1.8 mm. Body shape (Fig. 2A, B) of agriloid type; expanded thorax distinctly wider than abdominal segments, except for abdominal segment I wider than metathorax and terminal abdominal segments VIII–IX at least of same width as prothorax. Body light yellow or white; surface smooth, with insignificant long setae.

Head prognathous, mostly retracted into prothorax. Labrum (Fig. 2F) strongly transverse, gradually becoming narrower anteriorly, approximately 2× wider than long; anterior margin straight, with dense mircosetae; lateral margins distinctly arched; surface weakly convex, smooth, with four short setae at base. Anteclypeus membranose, oblong, strongly transverse, approximately 3× wider than long; surface smooth.

Epistome (Fig. 2F) weakly sclerotized, brown, semitranslucent, strongly transverse, approximately 5.2× wider than long; anterior margin emarginate in middle; anterior angles rounded; surface smooth, distinctly convex, with four sensilla in two groups situated in shallow, round depressions in middle, as known in other buprestid larvae. Mandibles triangular, black, strongly sclerotized; basolateral outer margin with a long seta; internal margin with based penicillum bearing dense, short setae.

Maxillae (Fig. 2F): cardo strongly transverse, well sclerotized in lateral parts, weakly sclerotized and semitranslucent in middle part; posterior margin distinctly wider than anterior; lateral parts each with two long setae. Stipes subquadrate, slightly sclerotized; apical margin with setae as long as basal palpomere, and with a long seta on internal parts. Mala elongate, narrowed apically; anterior margin with setae slightly longer than those on stipes.

Antennae situated in deep incision, two-segmented, subcylindrical; antennomere I slightly expanded apically, approximately 1.2× as long as antennomere II and distinctly thicker than antennomere II; surface glabrous except anterior margin with dense microsetae. Second antennomere with a long trichosensilla, approximately 1.6× as long as antennomere II, and bearing some short trichosensilla extending beyond sensory appendage and two palmate sensilla on the apex of second antennomere (Volkovitsh and Hawkeswood 1990). Prementum (Fig. 2G) subquadrate, 1.2× as long as wide; anterior margin weakly arcuate; anterior angles rounded; lateral margins subparallel, anterior parts weakly expanded; anterior surface with dense microsetae, posterior border of microsetal area M-shaped; posterior surface glabrous.

Prothorax (Fig. 2C, D) distinctly longer and wider than mesothorax and metathorax, widest in the middle, 1.5× as wide as meso- and metathorax; lateral margins arcuate; dorsal and ventral plates each with a longitudinal pronotal and prosternal grooves; anterior part of pronotal groove slightly wider than posterior, posterior part not bifurcated. Mesothorax as wide as metathorax. Thoracic spiracles on lateral parts of mesothorax. Thorax without legs.

Lateral parts of abdominal segments with sparse, long hairs. Abdominal segments I–IX subquadrate, slightly wider in middle. Lateral parts of segments I–VIII each with a pair of spiracles anteriorly; segments IX and X without spiracles. Posterior part of abdominal segment X rounded, lateral parts with long setae denser than in middle, with a pair of sclerotized terminal processes. Terminal processes long, subcylindrical, gradually tapering from base to apex; each process with two subdivisions in internal margin (Fig. 2E).

##### Description of pupa

**Figure 3.**
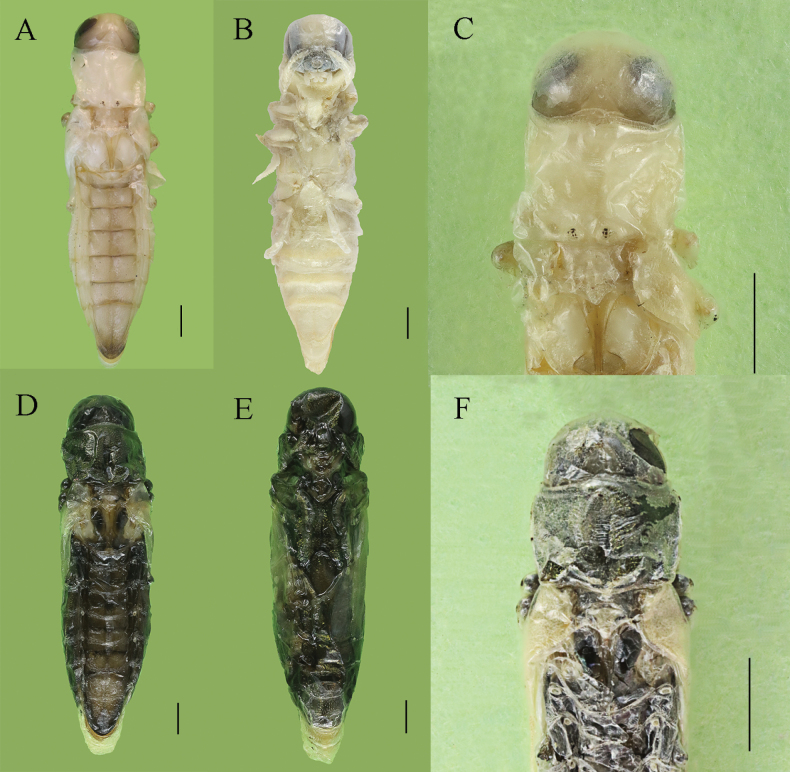
Pupa of *Agrilusadelphinus***A, D** dorsal view **B, E** ventral view **C, F** head and pronotum in dorsal view. Scale bars: 1 mm.

**(Fig. 3).** Body length 10–14 mm, width 3–3.8 mm. Body (Fig. 3A, B, D, E) exarate, white; eyes and mouthparts darker. Pygidium slightly brown apically; body surface smooth, without setae.

Head hypognathous; mouthparts and frons invisible in dorsal view; most eyes and vertex visible in dorsal view; surface with dense, small, black spots. Mandibles strongly sclerotized. Antennae placed along lateral sides of prosternum, directed backwards, reaching basal margin of prosternum.

Pronotum (Fig. 3C, F) shaped nearly like an inverted trapezoid, widest in anterior 1/3; anterior margin distinctly wider than posterior; anterior angles produced; anterior pronotal lobe arcuate and not reaching level of anterior angles; lateral margins weakly arcuate; posterior angles nearly rectangular; Posterior margin with two strongly convex tubercles in middle; disk smooth. Prosternal process narrowed; angles of prosternal process obtuse. Prehumerus carinal, posterior end joining posterior pronotal angle. Marginal and submarginal carinae converging and fused posteriorly, interspace wide, narrowest point at posterior 1/3 of pronotum. Mesonotum strongly impressed, except base of elytra. Elytra distinctly developed; elytral apex extending to posterior margin of abdominal ventrite III. Most part of metathoracic wings covered by elytra, extending to anterior margin of abdominal ventrite II. Metasternal projection impressed. Metanotum with a deep, longitudinal groove, nearly V-shaped, anterior part distinctly wider than posterior. Legs semitransparent.

Abdomen widest at tergites IV (ventrite I + II). Tergites I–VII with dense, large punctures bearing very short setae. Tergites I–VI subequal in length; pygidium distinctly longer than other tergites, posterior margin arcuate, with setae longer than those on tergites I–VI; anterior margin of tergites III–VI and posterior margin of tergite I black. Ventrite I + II distinctly longer than ventrites III–V; posterior margins of ventrites I + II to IV light brown. Surface of ventrites I– IV smooth, with indistinct short setae; posterior of ventrite V with long setae; posterior margin of sternite V arcuate. Spiracles located on anterio-lateral margin of tergites I–VII, paired, and ovate; spiracles on tergite I distinctly larger than those on tergites II– VII. Female: posterior margin of sternite V deeply, arcuately sinuate.

### ﻿Phylogenetic analyses

A total of 69 *COI* fragment sequences (including 10 new sequences) of 57 *Agrilus* species and two outgroup sequences of *Coraebus* Gory & Laporte, 1839 were used for phylogenetic analysis based on the best-fitting model GTR+F+I+G4.

**Figure 4.**
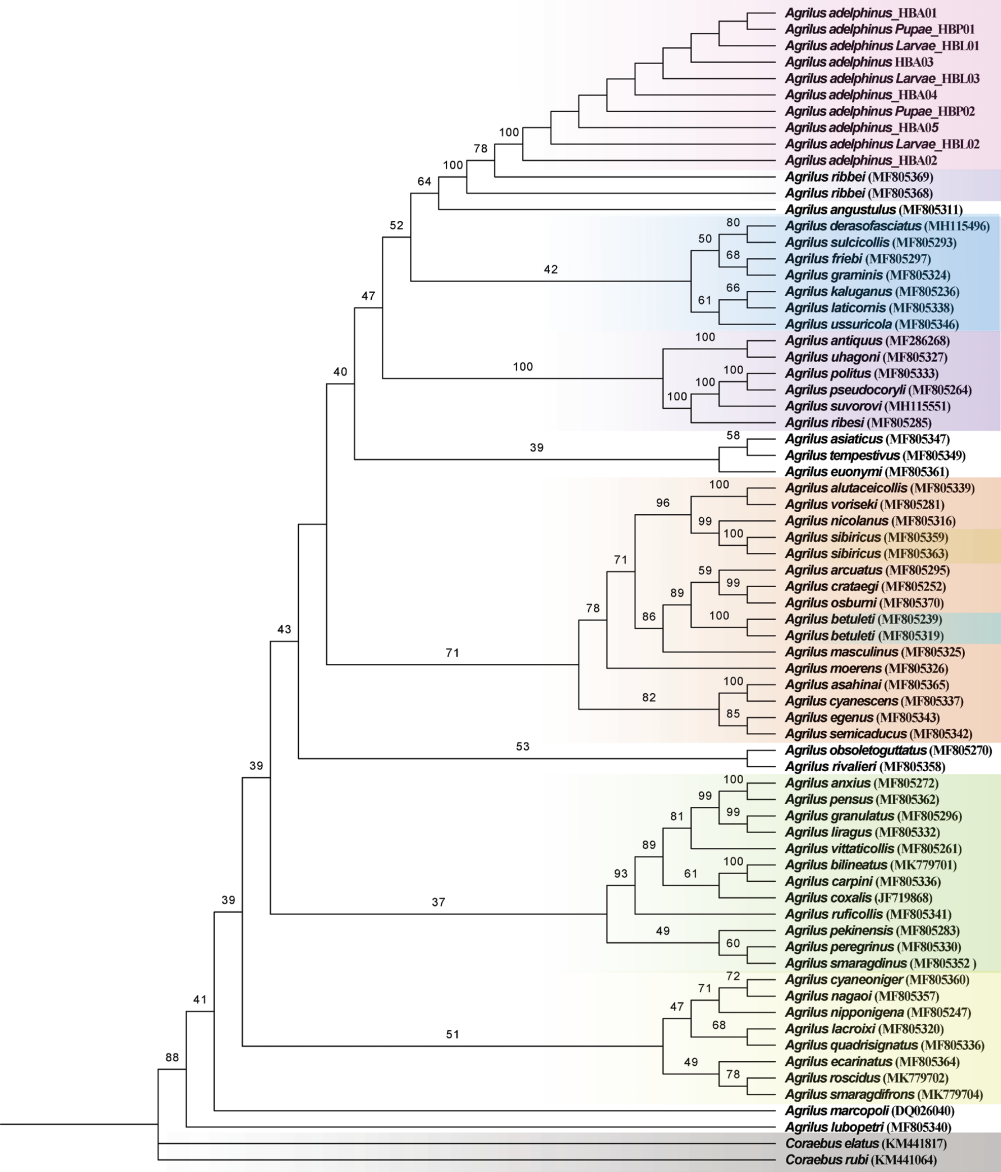
Maximum-likelihood (ML) tree of 57 *Agrilus* species based on 69 *COI* fragment sequences. ML bootstrap values are shown at each node.

In the ML tree, all species of *Agrilus* are separate from the outgroups, forming a large branch (Fig. 4). The results show that unknown larvae and pupae form a single, highly supported clade (ML bootstrap = 100) adults of *A.adelphinus*. Moreover, the target (((((((((*A.adelphinus*_HBA01 + *A.adelphinus* Pupae_HBP01) + *A.adelphinus* Larvae_HBL01) + *A.adelphinus*_HBA03) + *A.adelphinus* Larvae_HBL03) + *A.adelphinus* _HBA04) + *A.adelphinus* Pupae_HBP02) + *A.adelphinus* _HBA05) + *A.adelphinus* Larvae_HBL02) + *A.adelphinus*_HBA02) clade formed a large sister clade with the *A.ribbei* clade. The results show that larvae, pupae, and adults of *A.adelphinus* belong to the same species, and confirmed that *A.ribbei* is very closely related to *A.adelphinus* which is first demonstrated by Kelnarova et al. (2019). *Agrilusadelphinus* + *A.ribbei* form a subclade within the *A.sulcicollis* species-group or subgenus Quercuagrilus.

In addition to the same species forming a branch with 100 nodal support, there several other species clustered together, also having 100 nodal support. For example, the (*A.antiquus* + *A.uhagoni*) clade and the (((*A.politus* + *A.pseudocoryli*) + *A.suvorovi*) + *A.ribesi*) clade form a branch with 100 nodal support, which was first demonstrated by Kelnarova et al. (2019). Similarly, *A.alutaceicollis* and *A.voriseki* form a branch with 100 support value, *A.asahinai* and *A.cyanescens* also form a branch with a 100 support value. The results also suggest that subgenera and species-groups can be verified, revised, and improved based on phylogenic analyses.

We conclude that larvae and pupae which have the same *COI* fragment sequences as adults, undoubtedly belong to the same species, *A.adelphinus*.

### ﻿Mitogenome

#### Genome organization and base composition

**Figure 5.**
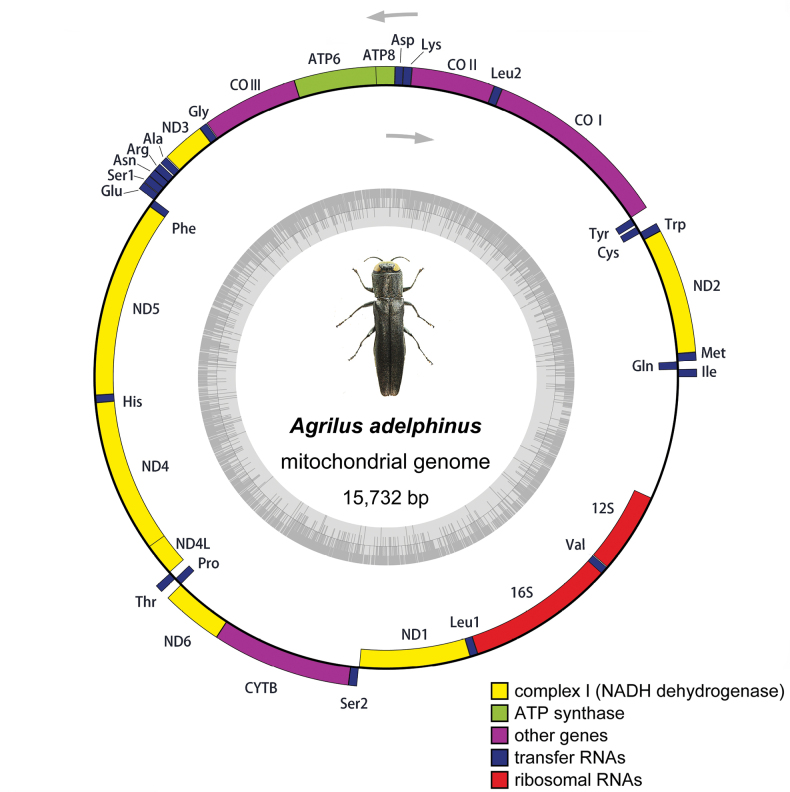
Gene maps of the complete mitogenome of *Agrilusadelphinus*.

The mitogenome extraction of *A.adelphinus* had a circular DNA molecule with 15,732 bp (GenBank accession no. OP401219; SRA accession no. SRR23527510). The circular map for this mitogenome is presented in Fig. 5. It is composed of a long non-coding A+T-rich region and 37 coding genes (22 tRNAs, 2rRNA, and 13 PCGs). Among these, four PCGs (*ND4L*, *ND4*, *ND5*, and *ND1*), eight tRNAs (*tRNA^Gln^*, *tRNA^Cys^*, *tRNA^Tyr^*, *tRNA^Phe^*, *tRNA^His^*, *tRNA^Pro^*, *tRNA^Leu1^*, and *tRNA^Val^*), and two rRNAs (*12S* and *16S*) are encoded on the N-strand. The other 23 genes (9 PCGs and 14 tRNAs) are encoded on the J-strand.

In this species, there are several small noncoding intergenic spacers in addition to the large noncoding A + T-rich region; these are usually made up of fewer than 10 non-coding nucleotides in the mitochondria of most animals (Podsiadlowski 2010). The total length of the 10 intergenic regions in the *A.adelphinus* mitogenome is 59 bp (Table 1), while longer than usual noncoding elements were found in the intergenic spacer region between the *tRNA^Cys^* and *tRNA^Tyr^* genes, the length of which is 18 bp. This spacer is the same length as in *Coomaniellacopipes* Jendek & Pham, 2013 (Huang et al. 2022) but in a different location. The unusual intergenic interval in *Trachysauricollis* Saunders, 1873 even had five locations (Sun et al. 2020). This spacer exists in many mitogenomes of Coleoptera and serves as a constant molecular marker of mitochondrial DNA of Coleoptera (Zhang et al. 2015). In the whole mitogenome of *A.adelphinus*, the length of the 13 overlapping regions was 37 bp, among which the maximum and minimum length of overlap was 8 bp located at one junction (*tRNA^Trp^* and *tRNA^Cys^*) and one bp located at eight junctions (*tRNA^Gln^* and *tRNA^Met^*, *ND2* and *tRNA^Trp^*, *ATP6* and *COIII*, *tRNA^Arg^* and *tRNA^Asn^*, *tRNA^Glu^* and *tRNA^Phe^*, *tRNA^Thr^* and *tRNA^Pro^*, *ND6* and *CYTB*, *CYTB*, and *tRNA^Ser2^*, respectively). For *ATP8*–*ATP6*, an atypical overlapping sequence of ATGATAG was identified. However, a typical ATGTTAA sequence could be observed between *ND4* and *ND4L*. Consistent with most studies, no gene rearrangement was found.

**Table 1.** Annotation and gene organization of the mitochondrial genome of *Agrilusadelphinus*.

Gene	Strand	Position		Codons		Anticodon	IGN
		From	To	Start	Stop		
*tRNA^Ile^*	J	1	67			GAT	–3
*tRNA^Gln^*	N	65	133			TTG	–1
*tRNA^Met^*	J	133	201			CAT	0
*ND2*	J	202	1224	ATT	TAA		–1
*tRNA^Trp^*	J	1224	1295			TCA	–8
*tRNA ^Cys^*	N	1288	1352			GCA	18
*tRNA^Tyr^*	N	1371	1436			GTA	7
*COI*	J	1443	2973	–	T(AA)		0
*tRNA^Leu2^*	J	2974	3041			TAA	0
*COII*	J	3042	3723	ATA	T(AA)		0
*tRNA^Lys^*	J	3724	3793			CTT	0
*tRNA^Asp^*	J	3794	3860			GTC	0
*ATP8*	J	3861	4019	ATC	TAG		–7
*ATP6*	J	4013	4687	ATG	TAA		–1
*COIII*	J	4687	5475	ATG	TAA		4
*tRNA^Gly^*	J	5479	5544			TCC	0
*ND3*	J	5545	5898	ATT	TAA		5
*tRNA^Ala^*	J	5904	5967			TGC	9
*tRNA^Arg^*	J	5977	6040			TCG	–1
*tRNA^Asn^*	J	6040	6104			GTT	0
*tRNA^Ser1^*	J	6105	6171			TCT	1
*tRNA^Glu^*	J	6,173	6,237			TTC	–1
*tRNA^Phe^*	N	6,237	6,301			GAA	0
*ND5*	N	6,302	8,024	ATA	T(AA)		0
*tRNA^His^*	N	8,025	8,089			GTG	0
*ND4*	N	8,090	9,425	ATG	T(AA)		–7
*ND4L*	N	9,419	9,703	ATG	TAA		6
*tRNA^Thr^*	J	9,710	9,776			TGT	–1
*tRNA^Pro^*	N	9,776	9,843			TGG	1
*ND6*	J	9,845	10,351	ATA	TAA		–1
*CYTB*	J	10,351	11,493	ATG	TAA		–1
*tRNA^Ser2^*	J	11,493	11,562			TGA	4
*ND1*	N	11,566	12,531	TTG	TAG		0
*tRNA^Leu1^*	N	12,532	12,599			TAG	0
*16S*	N	12,600	13,885				–5
*tRNA^Val^*	N	13,881	13,950			TAC	4
*12S*	N	13,955	14,661				0
A + T-rich region	J	14662	15,732				0

#### Protein-coding regions and codon usage

PCGs have the largest proportion in the *A.adelphinus* mitogenome sequence (11,173 bp, 71.02%, Table 2), but the A + T content is smaller than that of the whole (71.35%), rRNAs (76.12%), tRNAs (74.41%), and A + T-rich region (80.77%). In total, the 13 PCGs encoded 3,714 amino acids. Consistent with most studies, we also found that *ATP8* and *ND5* are the smallest and largest genes, respectively. Except for the *COI* gene with an undetermined start codon and the *ND1* gene starting with TTG, the remaining PCGs directly uses ATN (ATA/ATC/ATG/ATT) as the start codon. The majority of PCGs have complete termination codons (TAA/TAG), and only four PCGs (*COI*, *COII*, *ND5*, and *ND4*) have incomplete termination codons T-.

**Table 2.** Summarized mitogenomic characteristics of *Agrilusadelphinus*.

Species	PCGs			rRNAs			tRNAs Size(bp			A + T-rich region		
	Size (bp)	A+T (%)	AT skew	Size (bp)	A+T (%)	AT skew	Size (bp)	A+T (%)	AT skew	Size (bp)	A+T (%)	AT skew
*A.adelphinus*	11,173	69.19	–0.15	1993	76.12	–0.12	1477	74.41	0.0002	1071	80.77	0.06

**Figure 6.**
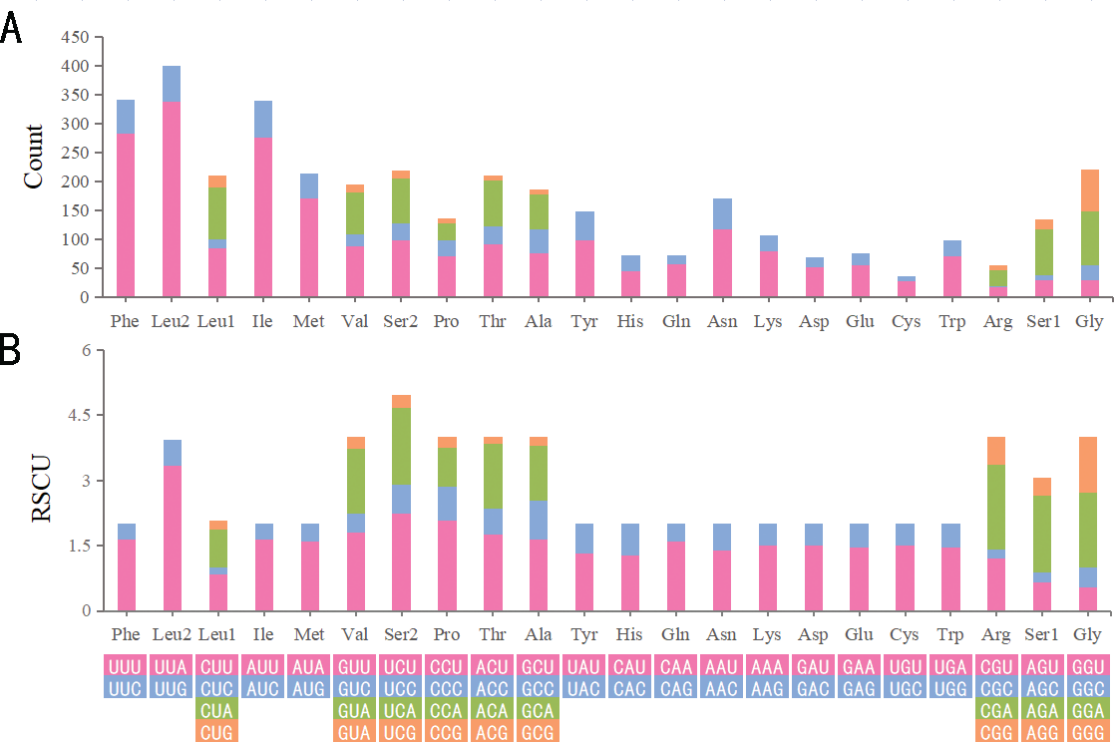
Protein-coding genes of the mitogenome of *Agrilusadelphinus***A** amino acids **B** relative synonymous codons.

The five most commonly encoded amino acids in the mitogenome of *A.adelphinus*, listed in order of decreasing frequency, are as follows: Leu2, Phe, Ile, Gly, and Ser2. The five most frequently used codons are: UUA (Leu2), UUU (Phe), AUU (Ile), AUA (Met), AAU (Asn) (Fig. 6). The preference of nucleotide composition in the mitogenome can be reflected by the use of codons. Correspondingly, we found that the RSCU in the mitogenome of *A.adelphinus* showed a strong preference for A and T, especially at the third codon position.

#### Transfer and ribosomal RNA genes

The 22 tRNA genes in the mitogenome of *A.adelphinus* are interspersed between the PCGs and rRNAs and range in size from 64 bp (*tRNA^Arg^*, *tRNA^Ala^*) to 72 bp (*tRNA^Trp^*) (Table 1). The total length, A + T content, and AT skew of the 22 tRNAs in the mitogenome of *A.adelphinus* are 1477 bp, 74.41%, and 0.0002, respectively (Table 2). All tRNAs have a typical secondary structure-cloverleaf shape, with the exception of *tRNA^Ser1^*, whose dihydrouridine (DHU) arm is missing, forming a simple loop (Suppl. material 1: fig. S1), which is the same as most insects (Hong et al. 2009; Li et al. 2012; Song et al. 2019; Xiao et al. 2019; Sun et al. 2020) and is considered to be a typical feature of insect mitogenomes (Lavrov et al. 2001; Stewart and Beckenbach 2006; Li et al. 2012). Not only that, some tRNAs also have a UG mismatch. The rRNAs are located between *tRNA^Leu1^* and A + T-rich region, separated by *tRNA^Val^*. The length of *16S* is 1286 bp, while the length of *12S* is 707 bp. The A + T content and AT skew of the two rRNAs in the mitogenome of *A.adelphinus* are 76.12% and –0.12, respectively.

## ﻿Discussion

### ﻿Notes on taxonomy

Among the Chinese *Agrilus*, only two larvae, *A.planipennis* and *A.mali*, have been described in detail. The larva of *A.adelphinus* is the third species described, which can be separated from *A.planipennis* by the following characters: (1) pronotal groove not bifurcated posteriorly; (2) posterior angles of abdominal segments not protruded laterally; and (3) abdominal segment VIII and IX slightly wider than segment VII.

### ﻿Molecular phylogenetics

To clarify the true identities of the larvae and pupae collected in the wild and verify the validity of DNA barcode for the identification of the species of *Agrilus*, we have constructed a phylogenetic tree of *COI* gene sequences of the larvae, pupae, and adults. In this study, all species of *Agrilus* are separated from *Coraebus*, forming a large branch in the tree. The monophyly of *Agrilus* is again confirmed. The results show that unknown larvae and pupae are combined with adults of *A.adelphinus* in a single, highly supported clade (ML bootstrap = 100). The different stages of same species group together, and several other species also group into highly supported branches. These species are very closely related, and some belong to the same species-group (Kelnarova et al. 2019). For example, the (*A.antiquus* + *A.uhagoni*) clade and the (((*A.politus* + *A.pseudocoryli*) + *A.suvorovi*) + *A.ribesi*) clade form a branch with a 100 nodal support value. In fact, these species belong to the *A.viridis* species-group. Similarly, *A.alutaceicollis* and *A.voriseki* belong to the *A.betuleti* species-group; *A.asahinai* and *A.cyanescens* belong to the *A.cyanescens* species-group; *A.adelphinus* and *A.ribbei* belong to *A.sulcicollis* species-group.

The taxonomy of such a large genus as *Agrilus* is still not clear. Even if species-groups are used to classify the existing *Agrilus* species, there are still a large number of species which have not been placed into a species-group. Therefore, more samples and molecular data are needed to address this problem.

The results of this study suggest that the unknown larva and pupa belong to the same species and confirms that *COI* barcode sequences are a valid molecular tool to associate unknown larvae and pupae with known adults. It is further proved that DNA barcode technology can remove the limitation of traditional taxonomy that cannot identify pre-adult developmental stages with adults.

### ﻿Mitochondrial genome

Compared to the known mitogenomes of Buprestidae, the newly sequenced genome is shorter. Consistent with the known complete mitogenomes of buprestid species (Hong et al. 2009; Duan et al. 2017; Cao and Wang 2019a, b; Xiao et al. 2019; Sun et al. 2020; Chen et al. 2021; Peng et al. 2021; Huang et al. 2022; Wei 2022; Wei et al. 2023), it is typically composed of 37 coding genes and a non-coding A + T-rich region. In fact, their orientation is the same as that of almost all known buprestid species (Hong et al. 2009; Duan et al. 2017; Cao and Wang 2019a, b; Xiao et al. 2019; Sun et al. 2020; Chen et al. 2021; Peng et al. 2021; Huang et al. 2022; Wei 2022; Wei et al. 2023). However, the interval between genes is differs rather widely between genera and even between species. This is also one of the main reasons for the differing sizes of whole mitogenomes in species. In the mitogenome of *A.adelphinus*, the ATGATAG sequence was observed between *ATP8* and *ATP6*, which is ATGATAA in many insect mitogenomes. The overlapping nucleotides of *ND4L*–*ND4* are conservative, consistent with the ATGTTAA of most species (Wang and Tang 2017). Most PCGs use ATN as the start codon, but the exception is the *ND1* gene, which starts with TTG. The unusual start codon of the *ND1* gene is found in the mitogenomes of some other insects, such as *Julodisvariolaris* (Pallas, 1771) (TTG) and *Liriomyzatrifolii* (Burgess, 1880) (GTG) (Yang et al. 2013; Wei et al. 2023). Similarly, the most of PCGs have complete stop codons, and only four PCGs (*COI*, *COII*, *ND5*, and *ND4*) have incomplete stop codons T-. Mitochondrial genes have incomplete stop codons, which are common in metazoans (Miya et al. 2001). The traditional explanation for this phenomenon is that the end of TAA is produced by post-transcriptional polyadenylation (Anderson et al. 1981; Deanna et al. 1981; Ojala et al. 1981). Unlike 21 other tRNAs with typical clover structure, *tRNA^Ser1^* lacks dihydrouridine (DHU) arm, which was the same as most other buprestid species.

This study provides new data on the phylogenetics of Buprestidae, improves our understanding of the mitogenome of *Agrilus*, and contributes to the further exploration of the relationships within the genus *Agrilus* and even the Buprestidae.

## Supplementary Material

XML Treatment for Agrilus adelphinus

## ﻿Additional information

### Author ORCIDs

Xuyan Huang https://orcid.org/0000-0003-2511-7080

Zhonghua Wei https://orcid.org/0000-0001-7349-9939

### Data availability

All of the data that support the findings of this study are available in the main text or Supplementary Information.

## Supplementary materials

Supplementary material 1 The larval, pupal and mitogenomic characteristics of *Agrilusadelphinus* (Coleoptera, Buprestidae) from China Data type table, images (word document) Explanation note The basic information of sequenced specimens in this study. The secondary cloverleaf structure for the tRNAs of *Agrilusadelphinus*. https://binary.pensoft.net/file/890085This dataset is made available under the Open Database License (http://opendatacommons.org/licenses/odbl/1.0/). The Open Database License (ODbL) is a license agreement intended to allow users to freely share, modify, and use this Dataset while maintaining this same freedom for others, provided that the original source and author(s) are credited.Xuyan Huang, Yujie Gan, Lei Wang, Yanying Xu, Zhonghua Wei, Aimin Shi
