# Supplementary material for: ﻿The larval, pupal and mitogenomic characteristics of Agrilusadelphinus Kerremans, 1895 (Coleoptera, Buprestidae) from China
Source: Zookeys. 2023 Aug 8;1174:15–33. doi: 10.3897/zookeys.1174.105479 (PMC10843335; doi:10.3897/zookeys.1174.105479)
Supplement: Supplementary material 1 — The larval, pupal and mitogenomic characteristics of Agrilusadelphinus (Coleoptera, Buprestidae) from China [file zookeys-1174-015_article-105479__-s001.doc]

**Table S1.** The basic information of sequenced specimens in this study.

| **No.** | **Taxa** | **GenBank** | **References** |
| --- | --- | --- | --- |
|  | *Agrilus adelphinus*_HBA01 | OP975841 | in this study |
|  | *Agrilus adelphinus*_HBA02 | OP975842 | in this study |
|  | *Agrilus adelphinus*_HBA03 | OP975843 | in this study |
|  | *Agrilus adelphinus*_HBA04 | OP975844 | in this study |
|  | *Agrilus adelphinus* _HBA05 | OP975921 | in this study |
|  | *Agrilus Larvae*_HBL01 | OP975920 | in this study |
|  | *Agrilus Larvae*_HBL02 | OP978140 | in this study |
|  | *Agrilus Larvae_*HBL03 | OP975916 | in this study |
|  | *Agrilus Pupae_*HBP01 | OP975845 | in this study |
|  | *Agrilus Pupae*_HBP02 | OP975917 | in this study |
|  | *Agrilus alutaceicollis* | MF805339 | Kelnarova et al., 2019 |
|  | *Agrilus angustulus* | MF805311 | Kelnarova et al., 2019 |
|  | *Agrilus antiquus* | MF286268 | Kelnarova et al., 2019 |
|  | *Agrilus anxius* | MF805272 | Kelnarova et al., 2019 |
|  | *Agrilus arcuatus* | MF805295 | Kelnarova et al., 2019 |
|  | *Agrilus asahinai* | MF805365 | Kelnarova et al., 2019 |
|  | *Agrilus asiaticus* | MF805347 | Kelnarova et al., 2019 |
|  | *Agrilus betuleti* | MF805239 | Kelnarova et al., 2019 |
|  | *Agrilus betuleti* | MF805319 | Kelnarova et al., 2019 |
|  | *Agrilus bilineatus* | MK779701 | Digirolomo et al.,2019 |
|  | *Agrilus carpini* | MF805366 | Kelnarova et al., 2019 |
|  | *Agrilus coxalis* | JF719868 | Coleman et al.,2012 |
|  | *Agrilus crataegi* | MF805252 | Kelnarova et al., 2019 |
|  | *Agrilus cyaneoniger* | MF805360 | Kelnarova et al., 2019 |
|  | *Agrilus cyanescens* | MF805337 | Kelnarova et al., 2019 |
|  | *Agrilus derasofasciatus* | MH115496 | Kolasam et al.,2019 |
|  | *Agrilus ecarinatus* | MF805364 | Kelnarova et al., 2019 |
|  | *Agrilus egenus* | MF805343 | Kelnarova et al., 2019 |
|  | *Agrilus euonymi* | MF805361 | Kelnarova et al., 2019 |
|  | *Agrilus friebi* | MF805297 | Kelnarova et al., 2019 |
|  | *Agrilus graminis* | MF805324 | Kelnarova et al., 2019 |
|  | *Agrilus granulatus* | MF805296 | Kelnarova et al., 2019 |
|  | *Agrilus kaluganus* | MF805236 | Kelnarova et al., 2019 |
|  | *Agrilus lacroixi* | MF805320 | Kelnarova et al., 2019 |
|  | *Agrilus laticornis* | MF805338 | Kelnarova et al., 2019 |
|  | *Agrilus liragus* | MF805332 | Kelnarova et al., 2019 |
|  | *Agrilus lubopetri* | MF805340 | Kelnarova et al., 2019 |
|  | *Agrilus marcopoli* | DQ026040 | Unpublished |
|  | *Agrilus masculinus* | MF805325 | Kelnarova et al., 2019 |
|  | *Agrilus moerens* | MF805326 | Kelnarova et al., 2019 |
|  | *Agrilus nagaoi* | MF805357 | Kelnarova et al., 2019 |
|  | *Agrilus nicolanus* | MF805316 | Kelnarova et al., 2019 |
|  | *Agrilus nipponigena* | MF805247 | Kelnarova et al., 2019 |
|  | *Agrilus obsoletoguttatus* | MF805270 | Kelnarova et al., 2019 |
|  | *Agrilus osburni* | MF805370 | Kelnarova et al., 2019 |
|  | *Agrilus pekinensis* | MF805283 | Kelnarova et al., 2019 |
|  | *Agrilus pensus* | MF805362 | Kelnarova et al., 2019 |
|  | *Agrilus peregrinus* | MF805330 | Kelnarova et al., 2019 |
|  | *Agrilus politus* | MF805333 | Kelnarova et al., 2019 |
|  | *Agrilus pseudocoryli* | MF805264 | Kelnarova et al., 2019 |
|  | *Agrilus quadrisignatus* | MF805336 | Kelnarova et al., 2019 |
|  | *Agrilus ribbei* | MF805368 | Kelnarova et al., 2019 |
|  | *Agrilus ribbei* | MF805369 | Kelnarova et al., 2019 |
|  | *Agrilus ribesi* | MF805285 | Kelnarova et al., 2019 |
|  | *Agrilus rivalieri* | MF805358 | Kelnarova et al., 2019 |
|  | *Agrilus roscidus* | MK779702 | Digirolomo et al.,2019 |
|  | *Agrilus ruficollis* | MF805341 | Kelnarova et al., 2019 |
|  | *Agrilus semicaducus* | MF805342 | Kelnarova et al., 2019 |
|  | *Agrilus sibiricus* | MF805359 | Kelnarova et al., 2019 |
|  | *Agrilus sibiricus* | MF805363 | Kelnarova et al., 2019 |
|  | *Agrilus smaragdifrons* | MK779704 | Digirolomo et al.,2019 |
|  | *Agrilus smaragdinus* | MF805352 | Kelnarova et al., 2019 |
|  | *Agrilus sulcicollis* | MF805293 | Kelnarova et al., 2019 |
|  | *Agrilus suvorovi* | MH115551 | Kolasa et al.,2019 |
|  | *Agrilus tempestivus* | MF805349 | Kelnarova et al., 2019 |
|  | *Agrilus uhagoni* | MF805327 | Kelnarova et al., 2019 |
|  | *Agrilus ussuricola* | MF805346 | Kelnarova et al., 2019 |
|  | *Agrilus vittaticollis* | MF805261 | Kelnarova et al., 2019 |
|  | *Agrilus voriseki* | MF805281 | Kelnarova et al., 2019 |
|  | *Coraebus rubi* | KM441064 | Hendrich et al., 2015 |
|  | *Coraebus elatus* | KM441817 | Hendrich et al., 2015 |


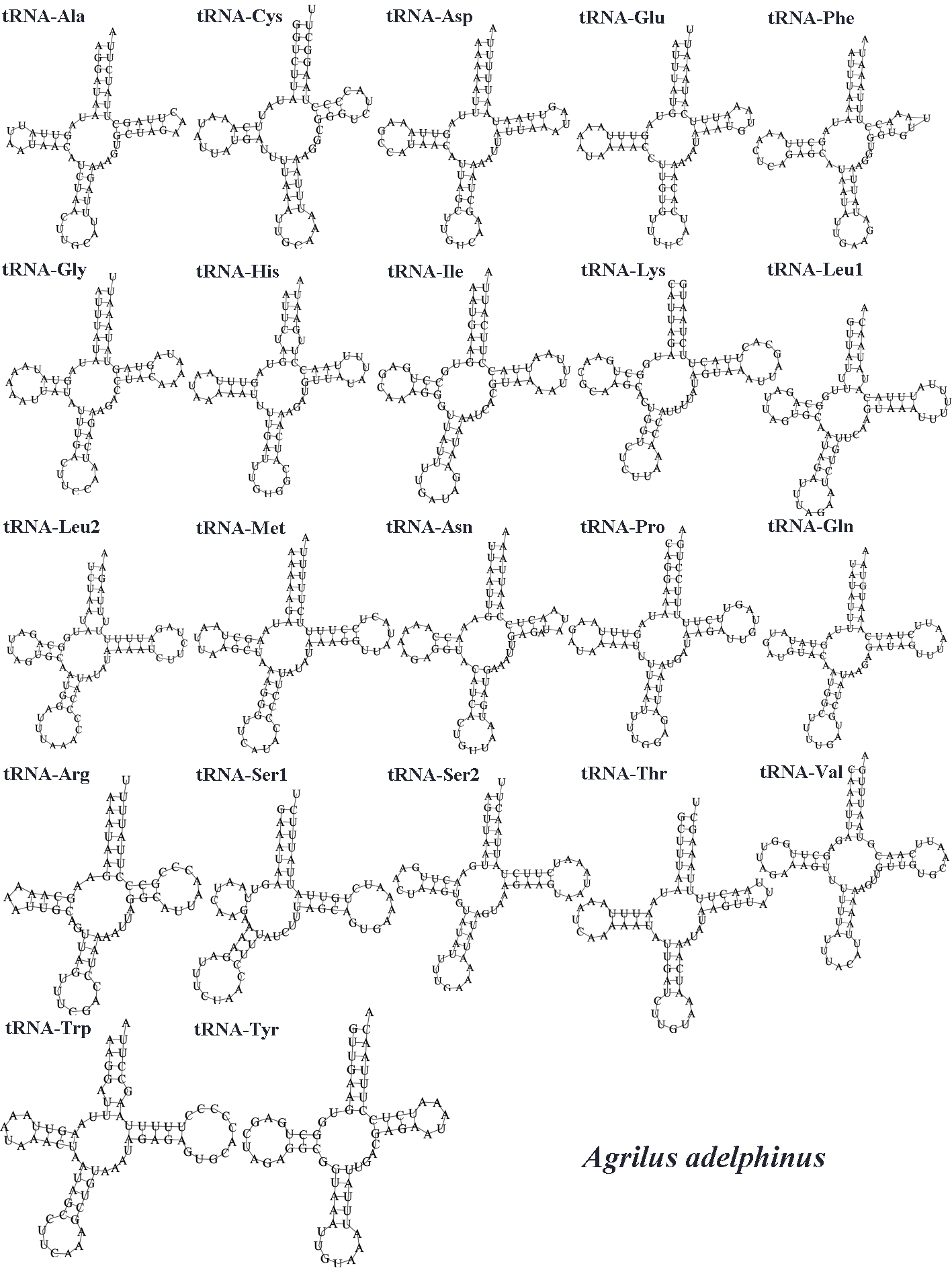


**Figure S1.** The secondary cloverleaf structure for the tRNAs of *Agrilus adelphinus*.

**Reference**

Kelnarova I, Jendek E, Grebennikov VV, Bocak L (2019) First molecular phylogeny of *Agrilus* (Coleoptera: Buprestidae), the largest genus on Earth, with DNA barcode database for forestry pest diagnostics. Bulletin of Entomological Research 109(2): 200–211.

Digirolomo MF, Jendek E, Grebennikov VV, Nakládal O (2019) First North American record of an unnamed West Palaearctic *Agrilus* (Coleoptera: Buprestidae) infesting European beech (*Fagus sylvatica*) in New York City, USA. European Journal of Entomology 116: 244–252.

Coleman TW, Lopez V, Rugman-Jones P (2012) Can the destruction of California’s oak woodlands be prevented? Potential for biological control of the goldspotted oak borer, Agrilus auroguttatus. BioControl 57: 211–225.

Kolasa M, Ścibior R, Mazur M.A, Kubisz D, Dudek K, Kajtoch Ł (2019) How Hosts Taxonomy, Trophy, and Endosymbionts Shape Microbiome Diversity in Beetles. Microbial Ecology. 78(4): 995–1013.

Hendrich L, Morinière J, Haszprunar G, Hebert PD, Hausmann A, Köhler F, Balke M (2015) A comprehensive DNA barcode database for Central European beetles with a focus on Germany: adding more than 3500 identified species to BOLD. Molecular Ecology Resources 15(4): 795–818.
